# Voltage divider effect for the improvement of variability and endurance of TaO_x_ memristor

**DOI:** 10.1038/srep20085

**Published:** 2016-02-02

**Authors:** Kyung Min Kim, J. Joshua Yang, John Paul Strachan, Emmanuelle Merced Grafals, Ning Ge, Noraica Davila Melendez, Zhiyong Li, R. Stanley Williams

**Affiliations:** 1 Hewlett Packard Labs, Hewlett Packard Enterprise, Palo Alto, California 94304, USA

## Abstract

The impact of a series resistor (R_S_) on the variability and endurance performance of memristor was studied in the TaO_x_ memristive system. A dynamic voltage divider between the R_S_ and memristor during both the set and the reset switching cycles can suppress the inherent irregularity of the voltage dropped on the memristor, resulting in a greatly reduced switching variability. By selecting the proper resistance value of R_S_ for the set and reset cycles respectively, we observed a dramatically improved endurance of the TaO_x_ memristor. Such a voltage divider effect can thus be critical for the memristor applications that require low variability, high endurance and fast speed.

Memristor is a promising electric component in future electronics due to its nonvolatile and reversible conductance change behavior^1^. Memristive devices which utilize the memristor have been intensively studied during the past decade for their potential applications such as digital storage^2^,^3^,^4^,^5^,^6^, analog computing^7^,^8^,^9^ and neuromorphic devices^10^,^11^,^12^. Despite of many promising properties, there are still a number of challenges against memristive devices for these applications. One of the major challenges is device endurance and variability^8^. It has been shown that the thermodynamic property of the memristive materials can significantly affect the device endurance and variability. Some materials, such as TaO_x_^5^,^13^,^14^,^15^,^16^,^17^,^18^,^19^ have exhibited smaller variability from switching cycle to cycle and thus greater endurance than other materials, such as TiO_x_^13^,^20^,^21^. This performance difference originates from the difference in thermodynamic properties of these two memristive materials. In order to have a stable switching system, i.e., small variability, the conduction channel (e.g. Ti_4_O_7_ in TiO_2_^22^ or TaO_x_ in Ta_2_O_5_^17^) needs to be thermally stable with the matrix materials (e.g. TiO_2_ or Ta_2_O_5_). This is true in the Ta-O system where there are only two thermodynamically stable solid phases but not in the Ti-O system where there are numerous suboxides^13^. Electrode materials have been shown to affect the variability as well. Chen *et al*. observed an improved endurance and variability by replacing the TiN electrode with Ru electrode in TaO_x_ memristors^19^, which may be explained by the formation of TiO_x_ at the TiN electrode surface.

In addition to the above approaches that focus on the material composition and structure, in this study we show that the variability issue can also be addressed by external control at the circuit level. The basic idea is to have sufficient voltage (>threshold voltage) to trigger the switching but to minimize any excess voltage applied on the memristive device during the switching. Considering the long tail in the lognormal distribution of the switching events^23^, memristive devices are typically overdriven by large switching voltages to minimize the ratio of unsuccessful switching events. Recently the so-called ‘self-limited switching behavior’ has been observed in a TaO_x_ device with an integrated series resistor (R_S_) that interrupts the over switching behavior automatically to enable more uniform set and reset switching^24^. This behavior is based on the voltage divider effect induced by the R_S_ component. Considering the role of the R_S_ in the self-limited switching as an excess voltage absorber, the presence of R_S_ may affect the variability and endurance performance of the memristor, which is elucidated in this study. We utilized discrete external resistors electrically connected to memristors because this allowed us to: 1) study the behavior of the same memristor with/without R_S_ and with R_S_ of different values, and 2) use two different series resistors for set and reset switching.

The TaO_x_ memristor has an inherently high endurance capability even without the R_S_ component when the switching is precisely controlled to minimize the over switching voltage. This can be achieved by using a sort of ‘incremental step pulse programming (ISPP)’ method which originated in a NAND flash memory and has been adopted in the memristor storage application^25^. Figure 1a shows the pulse sequences used in this test. In each writing process, i.e., the set switching that changes the memristor from the high resistance state (HRS) to the low resistance state (LRS), the writing voltage was increased gradually until the resistance value reached a target resistance value. In the erasing process, i.e., the reset switching that changes the memristor from the LRS to the HRS, a similar process was performed but using a negative voltage pulse. The target resistance values for the LRS and HRS were set to 2k ohm and 10k ohm, respectively. For this test, V_0_ and V_1_ were set to 1 V and −1 V, respectively, which were sufficiently low and did not induce any switching, and the ΔV was 0.1 V. Figure 1b shows the cycling data using this method for 10k cycles, where a clear resistance window can be seen. In this method, the programming attempts are repeated until the switching succeeds so that there is no error during the cycling. Although this programming method can guarantee 100% successful programming, it needs a much longer programming time and is not desirable for any applications that require high operation speed. During this cycling, the last programming voltage of each cycle was collected and the frequency distribution is shown in Fig. 1c. Both the set voltages (V_SET_) and reset voltages (V_RES_) displayed normal distributions, which is reasonable considering the random nature of the resistance switching. Here, those switching voltages in Fig. 1c can be regarded as the minimum switching voltages to get the target resistance values. In other words, for each programing event with just one voltage pulse, the switching voltages in absolute value should be larger than the maximum of the switching voltage distribution in order to guarantee a ~100% successful rate, e.g. the V_SET_ should be at least ~4.5 V according to the distribution of V_SET_ in Fig. 1c. However, at this so-called relaxed switching voltage condition, the excessive voltage leads to a greatly deteriorated cycling variability and endurance.

The switching endurance performance was investigated by using a single fixed switching voltage (to avoid the programing-read-programing verification sequence) for high speed operation. For this test, we developed a customized circuit board which could control the value of R_S_ during the pulse cycling to investigate the effect of R_S_. Figure 2a shows the schematic circuit configuration where the external R_S_ was connected in series with the memristor. The value of R_S_ can be set to 0, 500, 1k, and 2k ohms during the set and reset pulses independently. Therefore, by activating a specific value of R_S_ during the programming pulse and deactivating it during the read pulse, the exact resistance value of the memristor can be monitored, which enables a statistical analysis of the memristor resistance with respect to the R_S_. Figure 2b shows the pulsing sequence of voltage and R_S_ during cycling as a function of time. All of the pulse durations and intervals are 2 μs. Here, R_S,SET_ and R_S,RES_ refer to the values of R_S_ during the set and reset pulse duration, respectively, which are adjustable from 0 to 2k ohm. First, the endurance performance was investigated without the R_S,SET_. Here voltage conditions of −3.5 V for set and 4.0 V for reset were used, which were obtained from Fig. 1c. If the voltage amplitude was lower than those values, there would be a statistical chance of unsuccessful switching during cycling, which was avoided in this experiment. In this relaxed condition, the device was quite stable until ~250 cycles in terms of the read resistance. After ~250 cycles, however, the LRS of the device became more conductive and then the device finally went to an unrecoverable LRS after ~300 cycles which is known as the ‘set-stuck’ failure. The inset magnifies the cycling failure moment of the LRS. This is because of the high set switching stress which causes an irreversible damage to the device. Even though the switching was 100% successful before the failure, this set-stuck problem occurred quickly after a certain amount of cycles under the relaxed switching voltage condition. In this test, the R_S,RES_ was set to 0 ohm. The R_S,RES_ has little effect on the endurance with no R_S,SET_ because the set-stuck failure typically stems from the set switching.

When the R_S,SET_ was present, the endurance was significantly improved. However, another type of failure was observed. Figure 2d shows a typical endurance result with a non-zero R_S,SET_ and zero R_S,RES_. In this case, the endurance was much higher than the result of Fig. 2c because the R_S,SET_ can significantly suppress the set switching stress due to the voltage divider effect, which will be discussed later in detail. However, during the cycling, there was a statistical chance of immoderate reset switching that resulted in a very high resistance state. This higher resistance state actually does not cause the failure directly as the set switching does. However, the higher resistance state can result in a more drastic set switching with an increased set threshold voltage which accompanies a higher switching power and larger stress to the device. To illustrate this effect, Fig. 2e,f show plots of initial R_OFF_ versus the following R_ON_ after the set switching and the initial R_ON_ versus the following R_OFF_ after the reset switching, respectively. The colors in each plot show the threshold voltage for the set and reset switching. For this test, we used the pulse scheme as shown in Fig. 1a with 10k ohm of target resistance for both the R_ON_ and R_OFF_ where the goal is to see the wide range of resistance variations statistically. The results clearly show that a higher initial R_OFF_ needs a significantly higher set voltage, and results in a much lower R_LRS_. Similarly, a lower initial R_LRS_ needs a higher reset voltage and results in a much higher R_HRS_. Such a positive feedback behavior (higher R_HRS_ leads to lower R_LRS_, which in turn leads to higher R_HRS_ again) implies that the accidently obtained higher R_HRS_ may cause continuous set switching stress rather than a temporary stress. Thus, the higher R_HRS_ leads to a lower R_LRS_ which eventually resulted in the set-stuck failure as shown in Fig. 2d. Figure 2g schematically explains how the set-stuck happens under the set and reset stress conditions.

When both R_S,SET_ and R_S,RES_ are present, the aforementioned failures can be mitigated by the voltage divider effect and thus a reduced variability with enhanced endurance can be achieved. The voltage divider effect can be clearly observed in a DC measurement. Figure 3a shows the resistance – voltage (R – V) curves of the TaO_x_ memristor depending on the R_S_, ranging from 1k ohm to 2.5k ohm. The inset shows the measurement configuration. The R_ON_ is the sum of the R_S_ and the R_LRS_ (which is ~500 ohm in Fig. 2) of the memristor and was dominated by the R_S_ here. It can be seen that the reset threshold voltage increased with R_S_ because a higher voltage drops on the R_S_ as its resistance increases. Interestingly, the voltage divider effect was observed after the reset switching in the high voltage region. In the HRS, the R_HRS_ at the read voltage was much higher than the R_S_, thus it seems the voltage divider effect is insignificant for the reset switching. However, because the conduction resistance in the HRS is very nonlinear, which is a typical conduction characteristic of an insulating layer, the actual resistance at high voltages is not as large as the read resistance, which is clearly shown in Fig. 3a, e.g. the R_OFF_ at −4 V is only about 5k ohm while R_S_ was 1k ohm. Figure 3b shows the calculated node voltage of the memristor (V_M_, see the inset of Fig. 3a) depending on the R_S_ from Fig. 3a. A gray dashed line represents the case without the R_S_ for comparison (V_A_ = V_M_). When the R_S_ was involved in the HRS (arrow 1), the V_M_ was almost equal to the applied voltage (V_A_). Then, in the LRS after the set switching (arrow 2), only a relatively small portion of V_A_ dropped on the memristor and the V_M_ drastically decreased. At the negative voltage, as long as the memristor was in the LRS, the V_M_ stayed in very low value. Then, after the reset switching (arrow 3), the V_M_ drastically increased again close to the V_A_. An interesting observation to note here is that the V_M_ at the reset switching moment (black dashed line) is almost identical regardless of the R_S_. This confirms that increase of voltage drop across the R_S_ is responsible for the increase of the reset threshold voltage. Then the V_M_ in the HRS was saturated at −3 V due to the voltage divider effect reactivated in the high voltage region. Such voltage divider effects intervening in the high voltage portion of set and reset switching can suppress the high voltage stress on the memristor, playing a positive role on the variability and endurance performance. The impact of the voltage divider effect on the pulse switching was also investigated. For this measurement, the voltage and resistance pulsing system shown in Fig. 2a were used. Figure 3c shows the conductance (S = 1/R) dependence on the R_S_ as a function of the V_SET_ where the R_S,SET_ was changed from 0 (without R_S,SET_) to 5k ohm while the R_S,RES_ was fixed to 500 ohm which is the most stable reset condition to get the identical reset states. In this plot, the conductance was plotted instead of the resistance to clearly see the trace of the LRS. Each data point was obtained from 10k switching cycles by averaging the conductance values at each set voltage. The red X symbol indicates that the cycling failed before 10k cycles. In this case, only the successful cycling data were collected until the failure for this statistical analysis. When there was no R_S,SET_, the conductance change was very drastic with respect to an increase on V_SET_. Eventually the memristive switching quickly failed with a V_SET_ as low as 4 V. When the R_S,SET_ was presence, the increase of conductance in the LRS according to the increase of the V_SET_ can be suppressed up to 8 V of V_SET_. This clearly shows how the voltage divider effect works during the set switching in the pulse switching. Subsequently a similar behavior can be observed in the reset switching. Figure 3d shows the resistance dependence on the R_S,RES_ as a function of the V_RES_ where the R_S,RES_ was changed from 0 (without R_S,RES_) to 1k ohm while the R_S,SET_ was fixed to 500 ohm to prevent the set switching failure. When there was no R_S,RES_ during the reset switching, the highest resistance state can be reached at −3 V of V_RES_ and then the device failed at this condition before 10k cycles, which also shows the high sensitivity of the reset switching on the V_RES_ stress as discussed previously in Fig. 2d. When the V_S,RES_ was present, however, the memristor can be more endurable against the increase of V_RES_. As a result, the memristor can survive up to −5 V of V_RES_ with 1k ohm of R_S,RES_. When the R_S,RES_ was higher than 1k ohm, the reset switching was not observed until −5 V of V_RES_ because of the increase of reset threshold voltage as discussed in Fig. 1a^26^,^27^. So the applicable R_S,RES_ may not be as high as the R_S,SET_. The results shown in Fig. 3c,d both suggest that the voltage divider effect can reduce the high voltage stress on the memristor by absorbing the excess voltage.

When both the R_S,SET_ and R_S,RES_ were used, the aforementioned high set or reset stress can be eliminated. Therefore, the switching variability and endurance can be significantly improved. Figure 4a shows the cycling result with both the R_S,SET_ and R_S,RES_ for 1 million cycles where every single cycle was recorded with no error for the statistical analysis. Note that the endurance can be as high as 10^10^ cycles in this set-up^13^. Through the distributions of resistance states, the cause of endurance failure can be understood in the following. Figure 4b shows the LRS distributions collected from Fig. 2c (top panel), Fig. 2d (middle panel), and Fig. 4a (bottom panel), respectively. The R_LRS_ without the R_S,SET_ (top panel) has a symmetric distribution where many lower resistance states were observed (red square area) whereas the R_LRS_ with R_S,SET_ cases (middle and bottom panels) have a skewed distribution missing the lower resistance states in the distribution. Figure 4c shows the HRS distributions collected from Fig. 2c (top panel), Fig. 2d (middle panel), and Fig. 4a (bottom panel), respectively, where the resistance was plotted in a semi-log scale due to the wide resistance range in the HRS. Also a remarkable distribution tail was observed only in the case without the R_S,RES_ (blue square area in the middle panel) but not in the cases with the R_S,RES_ (top and bottom panels), which shows the impact of the voltage divider effect during the reset switching.

The external series resistors we adopted in this study allowed us to reveal the impact of the series resistors under different conditions by using external circuits. Based on the experimental results, we believe that an internal series resistor inside the memristive device may further enhance the role of a voltage divider due to a minimal parasitic resistance introduced to the circuit. Furthermore, by utilizing a transistor embedded in the memristive device, such as a transistor on a 1T1M or 2T1M configuration, both R_S,SET_ and R_S,RES_ can be tuned in real-time.

In conclusion, we demonstrated the effect of a series resistor on the variability and endurance of a TaO_x_ memristor. By intentionally tuning the R_S_ component during the pulse cycling, we have systematically shown that the presence and the resistance value tuning of R_S_ is critical for the improvement of memristor switching. Meanwhile, the Rs component does not cause any side effects on the performance of memristor. Therefore, we believe the Rs component may eventually be an indispensable component in future memristor circuits particularly for applications that require both low variability and fast programming speed.

## Experimental Procedure

### TaO_x_ memristor device fabrication

A 1×1 μm^2^ area of Pt/Ta/TaO_x_/Pt/Ta (from top to bottom) crossbar device was fabricated on a SiO_2_/Si substrate. The 10 nm Ta adhesive layer and 80 nm Pt bottom electrode were evaporated and patterned by the lift-off process. Then, a TaO_x_ layer was sputtered at 3 mTorr of Ar/O_2_ ambient using a Ta target. Finally, 10 nm Ta and 80 nm Pt top electrode were evaporated and patterned by the lift-off process.

### Electrical Measurements

DC electrical characterizations were performed using an Agilent 4156C Precision Semiconductor Parameter Analyzer. The pulse measurements were performed using a NI-7851R FPGA as the pulse generator in combination with a custom-made circuit board for reading the resistance of the memristor. During the electrical measurement, the top electrode was biased while the bottom electrode was grounded.

## Additional Information

**How to cite this article**: Kim, K. M. *et al*. Voltage divider effect for the improvement of variability and endurance of TaO_x_ memristor. *Sci. Rep.* **6**, 20085; doi: 10.1038/srep20085 (2016).

## Figures and Tables

**Figure 1:**
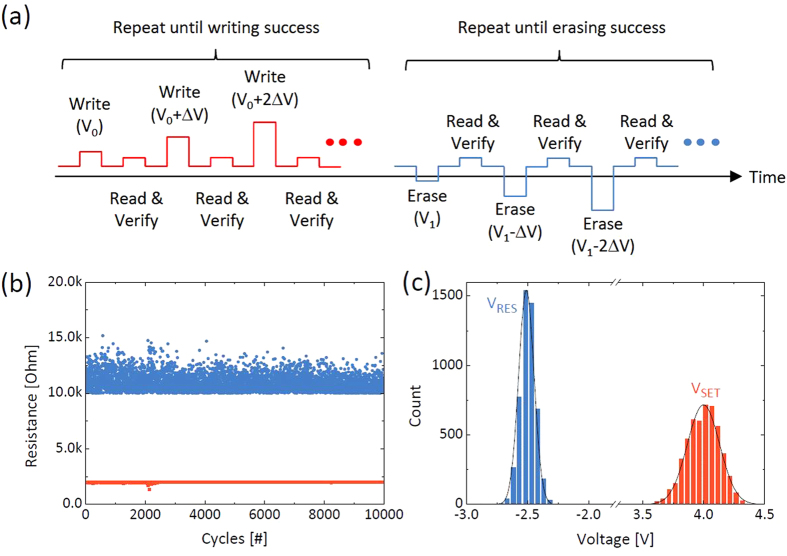
Pulse switching results of TaO_x_ memristor with the verification process. (**a**) A schematic of the incremental step pulse programming method used in this test, (**b**) The cycling result for 10k cycles with 2k and 10k ohm of target set and reset resistances, respectively. (**c**) The distributions of the switching voltages during this cycling.

**Figure 2:**
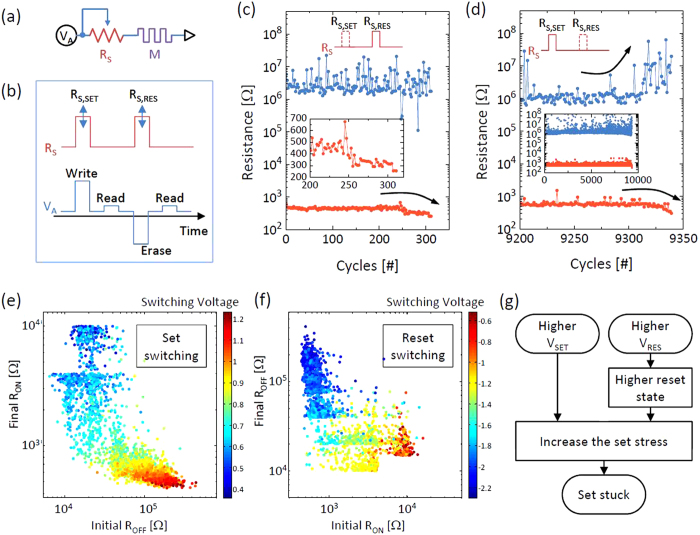
Pulse switching results without the verification process. (**a**) A schematic of the measurement set-up. (**b**) The pulse sequence of voltage and resistance as a function of time. The R_S,SET_ and R_S,RES_ are tunable among 0, 500, 1k, and 2k ohms. (**c**) The pulse switching result when the R_S,SET_ is disabled. (R_S,SET_ = 0, R_S,RES_ = 500 ohm). The inset magnified the trend of R_LRS_ at the set-stuck failure moment. (**d**) The magnified pulse switching result at the failure moment when the R_S,RES_ is disabled. (R_S,SET_ = 500 ohm, R_S,RES_ = 0). The inset shows the whole endurance characteristics. (**e,f**) The initial R_OFF_ and the followed R_ON_ (**e**) and the initial R_ON_ and the followed R_OFF_ (**f**) relations by the minimum set and reset switching conditions, respectively. The color scales represent the switching voltages during the resistance transition. (**g**) The flow chart shows the set-stuck failure process.

**Figure 3:**
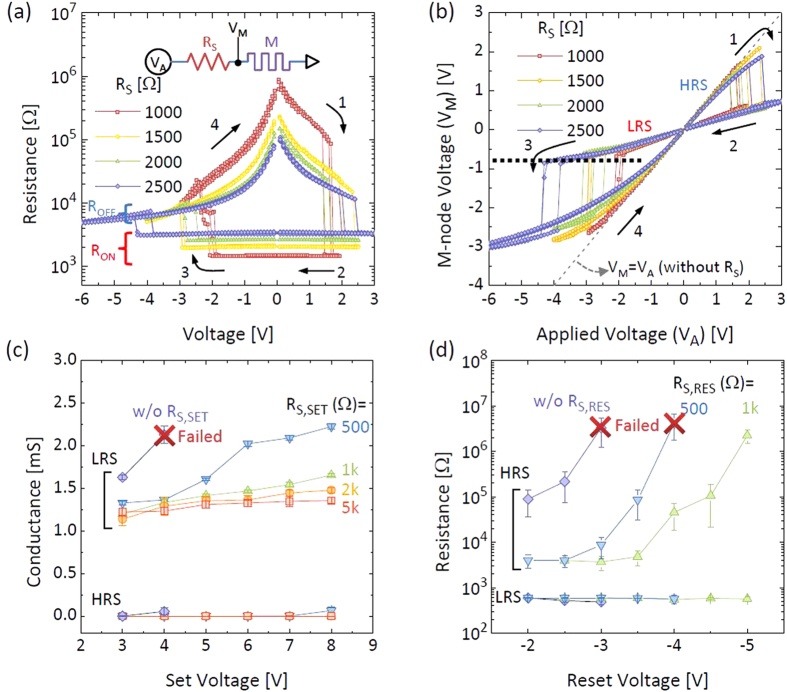
The voltage divider effect by the series resistor. (**a**) The DC R-V characteristics depending on the value of R_S_. The inset shows the measurement configuration. (**b**) The memristor node voltage (V_M_) as a function of the applied voltage (V_A_) depending on the value of R_S_. The dotted line indicates the identical V_M_ of the reset switching. (**c**) The conductance switching characteristics by the pulse as a function of the set pulse amplitude. The reset pulse was fixed to 4.5 V. (**d**) The resistance switching characteristics by the pulse as a function of the set pulse amplitude. The reset pulse was fixed to −3.0 V.

**Figure 4:**
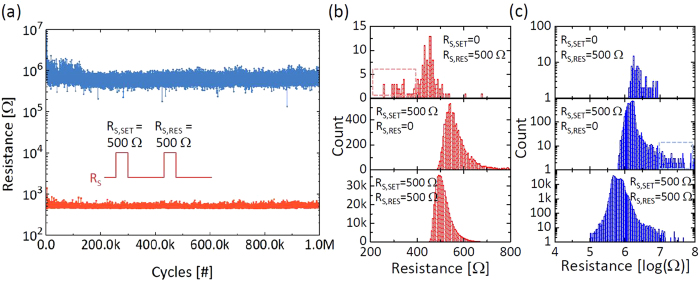
Low variability and high endurance pulse switching results with the series resistors. (**a**) A successful 10^6^ cycles switching without the verification process using −3.0 V and 4.5 V of set and reset pulses with 500 ohm of R_S_. (**b**) The LRS resistance distributions of Fig. 2c (top panel), Fig. 2d (middle panel), and Fig. 4a (bottom panel). (**c**) The HRS resistance distributions in log scale of Fig. 2c (top panel), Fig. 2d (middle panel), and Fig. 4a (bottom panel).
